# Letter

**DOI:** 10.1155/1992/63180

**Published:** 1992

**Authors:** Rifat Yalin, A. Ozdemir Aktan

**Affiliations:** Marmara University Hospital Dept. of General Surgery Istanbul Turkey


					HPB Surgery, 1992, Vol. 5, pp. 155
Reprints available directly from the publisher
Photocopying permitted by license only
Harwood Academic Publishers GmbH
Printed in the United Kingdom
LETTER
Dear Editor,
We read with great interest the paper "The postoperative appearances of the liver
on ultrasonography following hydatid cyst surgery" by Dr. liter and Dr. Mentes
which appeared in HPB Surgery 1990; 2: 253-260.
Although we are in agreement with the authors on most points, seyeral points
must be stressed.
In the postoperative follow-up of patients after hydatid cyst surgery, the major
problem is not the differentiation of the lesion from malignant lesions as no
association exists between hydatid cysts and malignant tumors. But the real
problem is to differentiate the normal postoperative image on ultrasonography
from recurrences. Recurrences, which must be expected in 10% of cases, and
undetected small cysts pose a great diagnostic problem. Hemaglutination test may
be helpful but there are false positive and false negative results and furthermore
they are not helpful in the postoperative period while seropositivity may continue
for up to six years.
We followed 24 patients after hydatid cyst surgery with both ultrasonography
(US) and computed tomography (CT). In a mean follow-up of 20 months (range 6-
72 months), there were four recurrences (1). In one of these US revealed no
pathology and in the remaining three CT was required to confirm the diagnosis.
We conclude that, while US is widely available and easy to use, the postoperative
follow-up of patients after hydatid cyst surgery may be misleading as normal images
may be regarded as recurrences and vice versa. CT is far more reliable in
demonstrating recurrences and the late course of the cystic cavity after surgery.
Sincerely yours.
1. Yalin, R., Aktan, A. O. Acikgozoglu, S. Computed tomography and sonography of hydated cysts
of the liver after surgical management. Journal of Medical Imaging 1989; 3: 301-305.
Rifat Yalin
A. Ozdemir Aktan
Marmara University Hospital
Dept. of General Surgery
Istanbul, Turkey
155

				

